# Using Internet and Mobile Phone Technology to Deliver an Automated Physical Activity Program: Randomized Controlled Trial

**DOI:** 10.2196/jmir.9.2.e7

**Published:** 2007-04-27

**Authors:** Robert Hurling, Michael Catt, Marco De Boni, Bruce William Fairley, Tina Hurst, Peter Murray, Alannah Richardson, Jaspreet Singh Sodhi

**Affiliations:** ^2^Tessella Support Services PLCAbingdonOxfordshireUK; ^1^Unilever Corporate ResearchColworthBedfordUK

**Keywords:** Behavior change, Health behavior, Behavior therapy, Obesity prevention, Health promotion, Exercise, Cellular phone, Internet, Consumer health informatics, Randomized controlled trial

## Abstract

**Background:**

The Internet has potential as a medium for health behavior change programs, but no controlled studies have yet evaluated the impact of a fully automated physical activity intervention over several months with real-time objective feedback from a monitor.

**Objective:**

The aim was to evaluate the impact of a physical activity program based on the Internet and mobile phone technology provided to individuals for 9 weeks.

**Methods:**

A single-center, randomized, stratified controlled trial was conducted from September to December 2005 in Bedfordshire, United Kingdom, with 77 healthy adults whose mean age was 40.4 years (SD = 7.6) and mean body mass index was 26.3 (SD = 3.4). Participants were randomized to a test group that had access to an Internet and mobile phone–based physical activity program (n = 47) or to a control group (n = 30) that received no support. The test group received tailored solutions for perceived barriers, a schedule to plan weekly exercise sessions with mobile phone and email reminders, a message board to share their experiences with others, and feedback on their level of physical activity. Both groups were issued a wrist-worn accelerometer to monitor their level of physical activity; only the test group received real-time feedback via the Internet. The main outcome measures were accelerometer data and self-report of physical activity.

**Results:**

At the end of the study period, the test group reported a significantly greater increase over baseline than did the control group for perceived control (*P* < .001) and intention/expectation to exercise (*P* < .001). Intent-to-treat analyses of both the accelerometer data (*P* = .02) and leisure time self-report data (*P* = .03) found a higher level of moderate physical activity in the test group. The average increase (over the control group) in accelerometer-measured moderate physical activity was 2 h 18 min per week. The test group also lost more percent body fat than the control group (test group: −2.18, SD = 0.59; control group: −0.17, SD = 0.81; *P* = .04).

**Conclusions:**

A fully automated Internet and mobile phone–based motivation and action support system can significantly increase and maintain the level of physical activity in healthy adults.

## Introduction

Physical inactivity is a major concern for developed societies. It accounts for about 12% of all deaths [1] and is associated with debilitating conditions [2]. In contrast, physical activity has been linked to positive health outcomes [3] and general well-being [4].

Government organizations recognize physical activity, along with a healthy diet, as playing an important role in the prevention of obesity [5], with a recommended level of moderate physical activity for adults of at least 30 min on most days of the week [6,7], with high-risk individuals benefiting from tailored interventions [8]. Unfortunately, infrequent exercise participation is common [9], starting even within late adolescence [10].

Internet-based behavioral change interventions minimize face-to-face interaction, thereby increasing cost-effectiveness through greater accessibility [11]. Partially automated programs, in which advice from therapists is delivered via email, can help people change health behaviors [12,13], and fully automated telephone counseling systems have also increased self-reported physical activity [14]. However, no research has evaluated the effect of a fully automated Internet-based system, with real-time objective feedback, on physical activity over several months [15]. Longer term studies using pedometers often rely on self-report [16].

Internet-based physical activity websites differ in their level of interaction, from individually tailored assistance to general guidelines or advice [17]. While more interactive elements (such as emailing weekly lessons) improve the number of people achieving health-related behavior change goals [13], it is still the case that Internet- and email-based systems can fail to hold participant interest [18]. A comparison of similar systems with different levels of interactivity found that the more interactive system was better able to retain participants [19].

We have developed a fully automated Internet, email, and mobile phone system [19] based on a range of social psychological theories (Social Comparison [20], Decisional Balance [21], Elaboration Likelihood [22], and Goal [23]). We used a Bluetooth [24] connected wrist-worn accelerometer to measure physical activity and provide feedback to participants.

Our primary hypothesis was that a group provided with access to the Internet and a mobile phone–based physical activity program would maintain a higher level of physical activity over 9 weeks than a control group who wore physical activity monitors but received no feedback and had no access.

## Methods

### Participants

**Figure 1:**
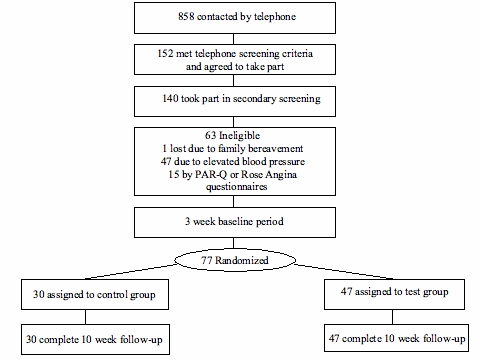
Flow diagram of study participation

A total of 140 people were initially recruited via a market research recruitment agency and passed the telephone screening (Figure 1) with self-report eligibility criteria as follows: age 30 to 55 years; body mass index (BMI) 19 to 30 (calculated from reported height and weight); not vigorously active; not taking regular prescription medication; Internet and email access; mobile phone user; and not employed by Unilever.

All participants agreed not to take part in any other studies, were briefed on the study aims, and signed an informed consent form in accordance with the Declaration of Helsinki [25]. Participants scoring one or more items on the Physical Activity Readiness Questionnaire (PAR-Q) [26] or the Rose Angina Questionnaire [27] were not accepted into the study and were provided with a letter to seek medical advice from their general practice doctor. For example, exclusion criteria from these questionnaires included the following: a heart condition, pain in the chest when exercising, and a joint problem that might be aggravated by exercise. In total, 77 healthy adults (51 women, 26 men) between 30 and 55 years (mean = 40.4 years; SD = 7.6) with a mean BMI of 26.3 (SD = 3.4) took part in the study, all living within 50 km of the study center in Sharnbrook, Bedfordshire, United Kingdom.

### Design

The 77 participants came to the center and were issued a wrist-worn accelerometer and Bluetooth-compatible mobile phone (Nokia 6230, with their SIM card inserted). After 3 weeks of monitoring baseline physical activity, participants returned and were stratified by age, gender, and BMI and were randomly allocated to either the control (n = 30) or test group (n = 47). More participants were allocated to the test group in order to maximize information on use of the system. All participants received £30 for attending the initial screening at the center, £140 to cover mobile phone costs, and £290 at closeout.

### Physical Activity Monitor

Although pedometers are low cost, they are typically attached to a waist band and therefore primarily record walking, making 24-hour monitoring more difficult. In contrast, accelerometry tools record a wider range of movement and have greater flexibility for body positioning, allowing for sustained monitoring even during sleep. Accelerometers have been widely used to monitor physical activity [28-30], both for school-age children [31] and adults [32].

Together with a technology company [33], we developed a Bluetooth wrist-worn device (Bluetooth Actiwatch) containing a miniature uniaxial accelerometer unit recording all movement over 0.05 g, excluding readings outside 3 to 11 Hz to eliminate gravitational artifacts. The signal was measured 32 times per second and digitally processed to integrate both the amount and duration of movement. Data were recorded with an epoch resolution of 2 min. The typical battery life was 6 months.

### Behavior Change System

**Figure 2:**
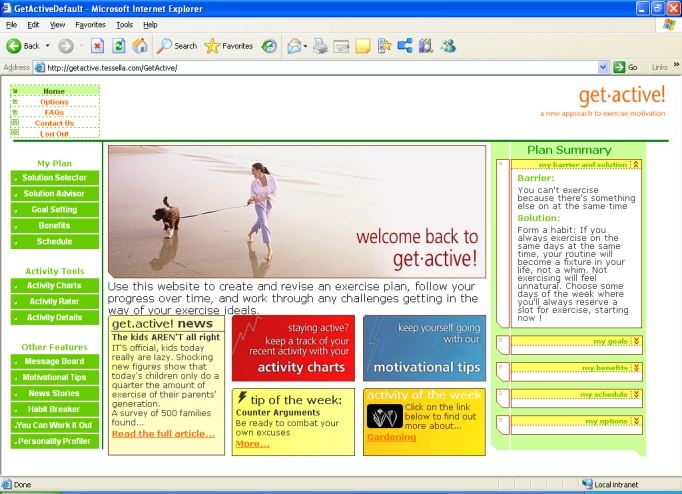
Behaviour change system home page

The Internet, email, and mobile phone behavior change system (Figure 2) was similar to that used in previous studies [19]. An introductory series of screens (Multimedia Appendix) helped test participants identify their perceived barriers to physical activity, offered solutions, and advised on appropriate wording for a commitment [34]. A weekly series of screens (see the Multimedia Appendix) asked the participants to report their exercise level during the last week, before providing constructive feedback on performance relative to their own target and the test group. The system included a weekly schedule (or diary) for planning physical activity sessions over the next 7 days (see the Multimedia Appendix), for which participants could choose to receive email and/or mobile phone reminders, an approach that has been effective in combination with implementation intentions [35]. The schedule included an automated “assessor” that provided feedback on the amount and type of physical activity being planned, advising a reduction in the case of participants planning to make very large increases compared with previous weeks.

A text-based automated dialogue module helped participants identify their perceived barriers and offered tailored solutions (see the Multimedia Appendix). For example, for the barrier “You can’t exercise because there’s something else on at the same time,” one of the solutions offered could be “Form a habit: If you always exercise on the same days at the same time, your routine will become a fixture in your life, not a whim. Not exercising will feel unnatural. Choose some days of the week where you’ll always reserve a slot for exercise, starting now!” Solutions were tailored to the individual via an underlying matrix that contained a strength of association between each barrier and solution. The strength of association between solutions and barriers increased in line with the increase in the level of physical activity of participants who had previously selected them.

**Figure 3:**
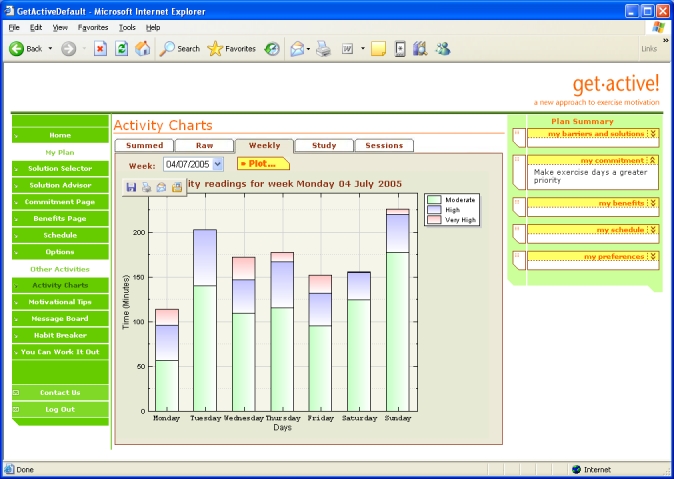
Weekly activity charts

Participants were also encouraged to select three motivating benefits, for which email and/or mobile phone text messages were optional. There was a library of information on a range of different physical activities, from household duties to team sports, and a chat-room style message board. Charts displayed real-time output from their physical activity monitor in three bands, moderate, high, and very high, with summaries for that day, the week (Figure 3), and the total study period, including the test group average. Low-level activity was excluded from the charts to reduce background noise. The system provided motivational tips matched to each participant’s current physical activity level. An automated dialogue therapy module helped people transform their rigid beliefs about exercise into more flexible, helpful beliefs [34,36]. The automated dialogue therapy module guided the participants through a series of steps, identifying a situation when a planned exercise was not carried out, identifying a “reason” the exercise was not carried out, explaining that this reason is actually a belief, and describing the difference between flexible and rigid (inflexible) beliefs, helping the participant create a more flexible belief to use next time he or she is in the same situation.

### Procedures

At the study center, participants received a full explanation about all procedures and were given an opportunity to ask questions. They were instructed to wear the Bluetooth Actiwatch on the wrist of their nondominant arm continuously for the following 12 weeks (3 weeks baseline and 9 weeks intervention). As the accelerometer was not fully waterproof, participants were asked to remove it when washing, bathing, or swimming. Following collection of 3 weeks of baseline data, the test group participants received a short demonstration of the Internet-based behavior change system; the control group also came to the center but only received verbal advice on recommended physical activity levels. The test group then had access to the Internet-based behavior change system for 9 weeks, whereas the control group had no access and received no feedback.

### Dependent Measures

The primary dependent measure was change in moderate physical activity recorded by the longer version of the International Physical Activity Questionnaire (IPAQ) [37] and the wrist-worn accelerometer. Changes in weight, percent body fat (as measured by bioelectrical impedance scales [38]), height [39], and resting blood pressure [40,41] were secondary measures. All measures were taken before and after the 9-week intervention period in September and December 2005, respectively.

A set of cognitive items was developed specifically for the study, each scored against a 7-point numbered scale ranging from “Strongly Disagree” to “Strongly Agree.” The items to measure motivation were as follows: “I am very satisfied with my level of fitness,” “I am very satisfied with my current level of motivation to exercise,” “I consider myself to be very healthy,” and “I am very happy about my general level of well-being” (Cronbach alpha = .89). The items to measure perceived control were “Exercise is too much effort” and “I feel in control of how much exercise I get” (Cronbach alpha = .63), and those measuring intention/expectation to exercise were “I intend to exercise for 30 minutes at least 3 times in the next week” and “I realistically expect that I will actually exercise for 30 minutes at least 3 times in the next week” (Cronbach alpha = .92). One item measured participant interest in using an Internet-based behavior change system: “I think an Internet-based motivation program could help people to take more exercise.” 

Participants also completed an exercise Skills and Knowledge Questionnaire that asked about skills used to increase physical activity [42]. Factor analysis indicated that participant responses fell into those related to their external environment (an external factor) and those related to internal motivation and confidence (an internal factor) [43]. The external factor items were “How confident are you that you know how to use prompts (reminders) to increase your physical activity,” “How confident are you that you know how to use rewards to increase your physical activity,” “How confident are you that you know how to get support from your family to increase your physical activity,” and “How confident are you that you know how to get support from your friends or colleagues to increase your physical activity” (Cronbach alpha = .89). The internal factor items were “How confident are you that you know how to set yourself achievable goals to increase your physical activity” and “How confident are you that you know how to make an action plan to increase your physical activity” (Cronbach alpha = .91).

### Statistical Analyses

Three participants were found to have faulty Actiwatches and so were removed from all statistical analyses. IPAQ data were processed according to the Guidelines for Data Processing and Analysis of the International Physical Activity Questionnaire [44]. We used an analysis of covariance model for the posttreatment data with pretreatment as a baseline covariate. A square-root transformation was applied to the data to protect against non-normality.

Actiwatch data for contiguous periods of zero extending longer than 30 min were omitted, as were data for periods with > 5000 counts for longer than 10 min since both these conditions indicated temporary malfunctioning of the accelerometer. Only days that had at least 10 h of recorded data following these corrections were retained for analysis [45]; of the 6634 eligible days of data, 177 were dropped (less than 3%).

A Generalized Estimating Equation Model with log link and Poisson distribution was used to calculate the number of 2-min epochs spent within three metabolic equivalent (MET) ranges [46], corresponding to moderate intensity (MET level over 3 and up to 6), high intensity (MET level over 6 and up to 9), and very high intensity (MET level over 9) (personal communication, S Brage, MRC Epidemiology Unit, Strangeways Research Laboratories, Cambridge, UK, 2005), during each individual’s waking day (identified from the 24-h Actiwatch data). Data points were only counted if they were part of a continuous bout of exercise of at least 10 min within the MET range. We corrected for baseline activity levels and week of study, but not for the length of day, as we were encouraging people to be more active, irrespective of the length of time they were awake. In order to represent the underlying signal, the data were smoothed using a moving average filter of width ± 1 point. Modifying the width of the filter had little effect on the results of the analysis.

We focused on the difference in total time of nonsedentary physical activity between the two groups rather than the absolute amount of physical activity within each group, as estimates of the latter can vary by a factor of 10 depending on the threshold point used [47].

Participants were instructed to remove the accelerometer for swimming—an activity selected by 36% of the test group who logged on. Therefore, our accelerometer-based estimate of physical activity did not fully account for all exercise undertaken, potentially attenuating any differences observed between the test and control groups. Anthropometric measures at the end of the study were analyzed using normal analysis of covariance models with baseline prestudy values as covariate. All analysis was carried out using SAS, version 9.1.3 [48].

## Results

A preliminary analysis showed that there were no differences between groups for baseline measures of age, weight, BMI, percent body fat, blood pressure, or initial level of physical activity, whether measured by the Actiwatch or IPAQ (Table 1).

**Table 1:** Baseline characteristics of participants

**Variable**	Test Group^*^(n = 47)	Control Group^*^(n = 30)	***P* value^†^**
Women (%)	64	70	.63
White ethnicity (%)	100	97	.39
Household broadband access (%)	29	22	.43
Age (years)	40.5 (7.1)	40.1 (7.7)	.97
Weight (kg)	75.1 (11.7)	73.9 (10.2)	.60
Height (cm)	166.3 (6.6)	165.2 (7.7)	.38
BMI	26.2 (2.8)	26.5 (4.1)	.68
Percent body fat (%)	30.2 (6.5)	31.0 (10.1)	.52
Blood pressure (mmHg) Systolic	119.8 (7.7)	118.2 (8.4)	.40
Blood pressure (mmHg) Diastolic	78.3 (5.7)	77.9 (6.1)	.82
Actiwatch accelerometer-measured time (epochs) spent above 3 and up to 6 METs during 3-week initiation period	228.0 (52.1)	214 .2 (53.1)	.11
Initial IPAQ self-report level of physical activity (MET mins)	4350 (3200)	3868 (2257)	.44

^*^Values are expressed as mean (SD) except for the first three variables.

^†^ *P* value is the probability that the two groups differ.

### Website Usage (Test Group Only)

More than 85% (mean = 86.4%, SD = 2.1) of test participants logged on each week during the first 4 weeks, decreasing to a plateau around 75% (mean = 76.1%, SD = 5.1) for the last 5 weeks. This level is lower than for partially automated behavior change systems [12] but higher than for other minimal-contact interventions [49,50].

The average number of log-ons per week was 2.9 (SD = 0.5), with short average duration of 7.5 min (SD = 0.9). The most frequently used components of the system were the activity charts (showing the accelerometer feedback data), the schedule (weekly exercise planner), and chat-room style message board. All components of the system were accessed by at least 33% of the participants during the intervention period. Typically, participants quickly formed an idiosyncratic preference for a few components of the system and then repeatedly used these throughout the intervention.

Comments on the message board indicated that participants found the system both educational and motivational, for example, “I am amazed looking at the graphs sometimes — I took my little fella to Bezerks in Northampton on Thursday morning and the graph went crazy with all the running around I did!”

The most popular (frequent) benefits of exercise were “Exercise will help me with weight loss” (n = 19), “I will have more energy” (n = 13), and “I will improve my muscle tone” (n = 11). The most commonly selected barriers to physical activity were time conflicts (n = 27), low motivation (n = 11), and procrastinating (n = 6).

### Self-Reported Changes in Physical Activity

As shown in Table 2, an intent-to-treat analysis of (the square-root transformed) MET minutes per week found no significant difference, after adjusting for the baseline covariate, between the test group (mean = 12.0, SE = 3.1) and the control (mean = 4.0, SE = 4.1), with *P* = .12 (95% CI for the difference = −2.3-18.3). When considering only MET minutes per week within leisure time, the increase in the test group was significantly higher than the control. The reduction in weekly hours spent sitting in the test group was significantly different from the control. There was a similar trend when breaking the data down into weekday sitting and weekend sitting (Table 2).

**Table 2:** Self-reported physical activity in test and control groups

**Self-Reported Physical Activity Variable**	Test Group^*^(n = 47)	Control Group^*^(n = 30)	***P* value^†^**	**95% CI for Difference**
**MET min/week**				
Overall	12.0 (3.1)	4.0 (4.1)	.12	−2.3 to 18.3
Leisure time	4.1 (2.6)	−5.5 (3.5)	.03	0.8 to 18.3
**Change in weekly hours spent sitting**				
Overall	−5.9 (2.0)	1.4 (2.7)	.03	−14.0 to −0.5
Weekday	−5.2 (1.7)	−0.2 (2.3)	.08	−10.8 to 0.7
Weekend	−0.9 (0.6)	1.2 (0.8)	.04	−4.2 to −0.1

^*^Values expressed as mean (SD).

^†^ *P* value is the probability that the two groups differ.

### Accelerometer Data

 We collected 4811124 data points from study participants, which represented 94.1% of the total expected based on the first and last recorded points for each individual (5114431).

**Figure 4:**
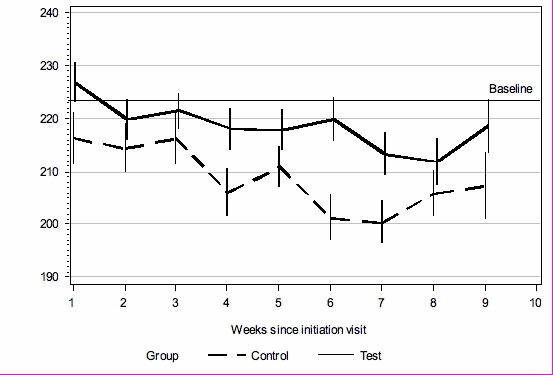
Accelerometer-measured mean number of 2-min epochs spent in moderate intensity MET range (above 3 and up to 6); error bars represent SE; baseline is 3-week average before start of intervention

Average sleep times for the two groups were not significantly different (test group: 467 min, SD = 40; control group: 468 min, SD = 38; *P* = .92). There was a significant trend over the whole study period indicating more time spent in the 3 to 6 MET range (moderate physical activity, eg, brisk walking) for the test group (log scale mean = 5.39, SE = 0.01) versus the control group (log scale mean = 5.34, SE = 0.01) with *P* = *.*02 (95% CI for the difference = 0.01-0.08). In the original units, this represents 218.5 epochs for the test group and 208.7 epochs for the control group, a difference of 19.7 min/day on average. The test group was consistently higher than the control group (in the moderate activity range) across all weeks of the study (Figure 4). Hence, the accelerometer data corroborated the significantly greater increase in physical activity self-reported by the test group. There was no significant difference between the groups in the ranges above 6 METs (log scale mean test group = 3.87, SE = 0.05; log scale mean control = 3.86, SE = 0.06; *P* = .94).

### Cognitive Measures

At the end of the study period, the test group reported a significantly greater increase over baseline than did the control group for the perceived control factor (mean change test group = +0.57; mean change control = −0.37; *P* < .001) and intention/expectation to exercise factor (mean change test group = +0.45; mean change control = −0.01; *P* < .001), but there was no significant difference in the motivational change factor (mean change test group = +0.11; mean change control = −0.02; *P* = .56). However, there were differences at the level of individual items within the factor. The test group rated themselves as more satisfied with their fitness (test mean = 3.94; control mean = 3.25; *P* = .047; final ratings corrected for baseline) and well-being (test mean = 5.04; control mean = 4.01; *P* = .007), a noteworthy result as changes in self-related measures of health have been related to objective health outcomes [51].

The Skills and Knowledge Questionnaire indicated that, after adjusting for baseline, the test group had a significantly higher sense of internal control (test mean = 7.24; control mean = 5.85; *P* = .003) and external control (test mean = 6.38; control mean = 5.33; *P* = .01) over exercise than did the control group at the end of the study period.

After the study period, the test group had a significantly greater interest in using an Internet-based behavior change system than the control group (test group = 4.92; control = 3.85; *P* < .001), indicating that test group participants had a positive experience.

### Anthropometry

The difference between the change in the test group’s BMI (mean change = −0.24, SE = 0.11) over the study period and that for the control group (mean change = 0.10, SE = 0.14) approached significance (*P* = *.*06; 95% CI = −0.02-0.70). The difference between the change in the test group’s percent body fat (mean change = −2.18%, SE = 0.59) over the study period and that for the control group (mean change = −0.17%, SE = 0.81) was statistically significant (*P* = .04; 95% CI = 0.06-3.94). There was no significant change in either diastolic (mean change test = 0.69, SE = 1.14; control = 0.73, SE = 1.49; *P* = .98) or systolic (mean change test = 0.13, SE = 1.33; control = 0.41, SE = 1.71; *P* = .90) blood pressure.

## Discussion

Increasing physical activity in the general population has an important role in the prevention of obesity and associated health problems [15,52]. We have shown that physical activity can be increased via a fully automated Internet-based behavior change system. The capture of real-time accelerometer data over 9 weeks while participants went about their everyday lives is in itself advancement for the field. Due to the use of Bluetooth technology, we were able to promptly detect and resolve most malfunctions in the accelerometers, resulting in extremely low data loss (< 10%). Access to the system was encouraging, with more than 70% of participants continuing to log on at least twice a week for all 9 weeks of the study. We attribute this to the interactive nature of the system we have developed [19]. It is essential that automated systems engage people in order for the behavior change program to have an impact [53]. As we only compared our system with a control group who received verbal advice, we cannot conclude that it would be superior to other interventions [54].

Although we observed an increase in accelerometer-measured physical activity for the test group over the control group, our analysis was limited by its uniaxial nature; future studies could employ a triaxial accelerometer so that greater differentiation of physical activity types can be achieved [55]. Also, since our accelerometers were only splash-proof, we were not able to capture physical activity for water sports.

The difference between the test and control group accelerometer-measured physical activity was apparent for most of the 9-week intervention (see Figure 4). The control group began at the same level as the test group but then decreased to a greater extent. It is likely that both test and control groups had initially higher levels of physical activity than their norm, due to awareness of being monitored and/or completing the questionnaires [56]. This suggests that the Internet-based behavior change system enabled the test group to maintain their elevated level of physical activity. The size of the difference in physical activity between the two groups is considerable; an increase of 2 h 18 min per week represents 92% of the recommended [6] 2 h 30 min, although further work is required to clarify how absolute continuous accelerometry measurements relate to the 30 min/day government recommendation.

In line with the Theory of Planned Behavior [57], which has been widely applied to a range of health behaviors [58], we found that the more physically active test group also reported a greater perceived control over their exercise behavior and greater intention/expectation to exercise. The test group level of motivation was not significantly different from the control, indicating that the intervention primarily influenced volitional aspects of behavior [23]. Unmotivated groups may require additional modules encouraging them to engage in the target behavior [59,60].

It was clear from the verbatim comments posted by participants on the message board that the accelerometer-based activity charts acted as educational information, allowing them to link periods of high physical activity to events in their everyday life. Indeed, everyday physical activities such as walking are considered to have the greatest potential for increasing overall activity levels of a sedentary population [61], and greater awareness of personal activity levels may lead to more positive intentions [62,63]. Unfortunately, a deficiency of our study is that we did not collect sufficient qualitative data to make a thorough analysis of how participants perceived the system.

In line with other research [64,65], we found BMI a less sensitive measure for physical activity interventions. However, the test group lost significantly more percent body fat in comparison to the control group, indicating that the increased physical activity may have led to greater muscle mass. An Internet-based behavior change system that improved diet as well as increasing physical activity may lead to more substantial losses of body fat and reduced BMI [66].

Most participants selected from a relatively small set of barriers and were motivated by similar benefits, as has been reported by other researchers [67]. Further work is required to explore how other groups of potential users react to the system and whether greater personalization is required [68].

Based on a range of behavior change principles taken from the literature [20,23,69], we included many different processes within our system, and so it is hard to determine which where the most effective in helping participants to change their behavior. The most popular parts of the system were the activity charts (showing the accelerometer feedback), schedule (weekly exercise planner), and chat-room style message board. The activity charts provided participants with feedback on their performance, which may have increased awareness and acted as a motivational spur for change, in line with Goal Theory [23]. The schedule can be considered a tool for making highly specific implementation intentions, which other research has shown to be an effective intervention for behavior change [59]. The message board could be considered a modern day representation of subjective norm (social pressure), as described within the Theory of Planned Behavior [57]. However, popularity (in terms of frequency of use) does not necessarily imply greater efficacy for behavior change. It is also notable that all parts of the system were used by at least one third of participants; it may be the case that each individual requires an idiosyncratic selection of support tools to achieve behavior change such that no one tool can be universally considered the most influential. Further work is required to determine how parts of the system interact to impact individual behavior change and how to optimize the exposure period; 9 weeks may not be necessary [70].

In summary, we found that participants with access to a fully automated behavior change system engaged in, on average, 2 h 18 min more physical activity per week than those with no access.

## Multimedia Appendix

Screenshots of the behaviour change system (ppt)
						

## Abbreviations

BMI: body mass index
IPAQ: International Physical Activity Questionnaire
MET: metabolic equivalent
