# Supplementary material for: Using Internet and Mobile Phone Technology to Deliver an Automated Physical Activity Program: Randomized Controlled Trial
Source: J Med Internet Res. 2007 Apr 27;9(2):e7. doi: 10.2196/jmir.9.2.e7 (PMC1874722; doi:10.2196/jmir.9.2.e7)
Supplement: Supplementary file 1 [file jmir_v9i2e7_app1.ppt]

## Slide 1
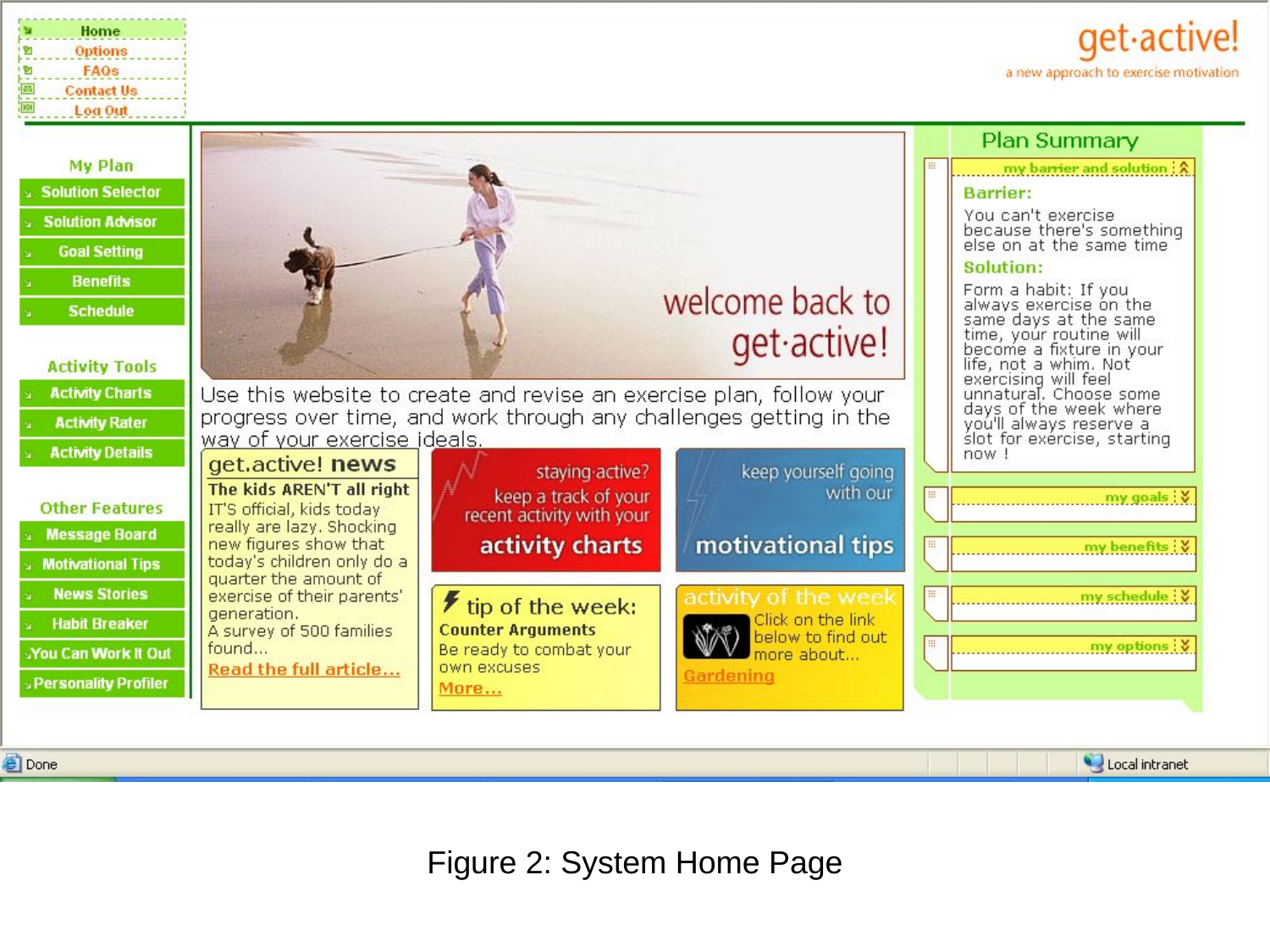

Figure 2: System Home Page

## Slide 2
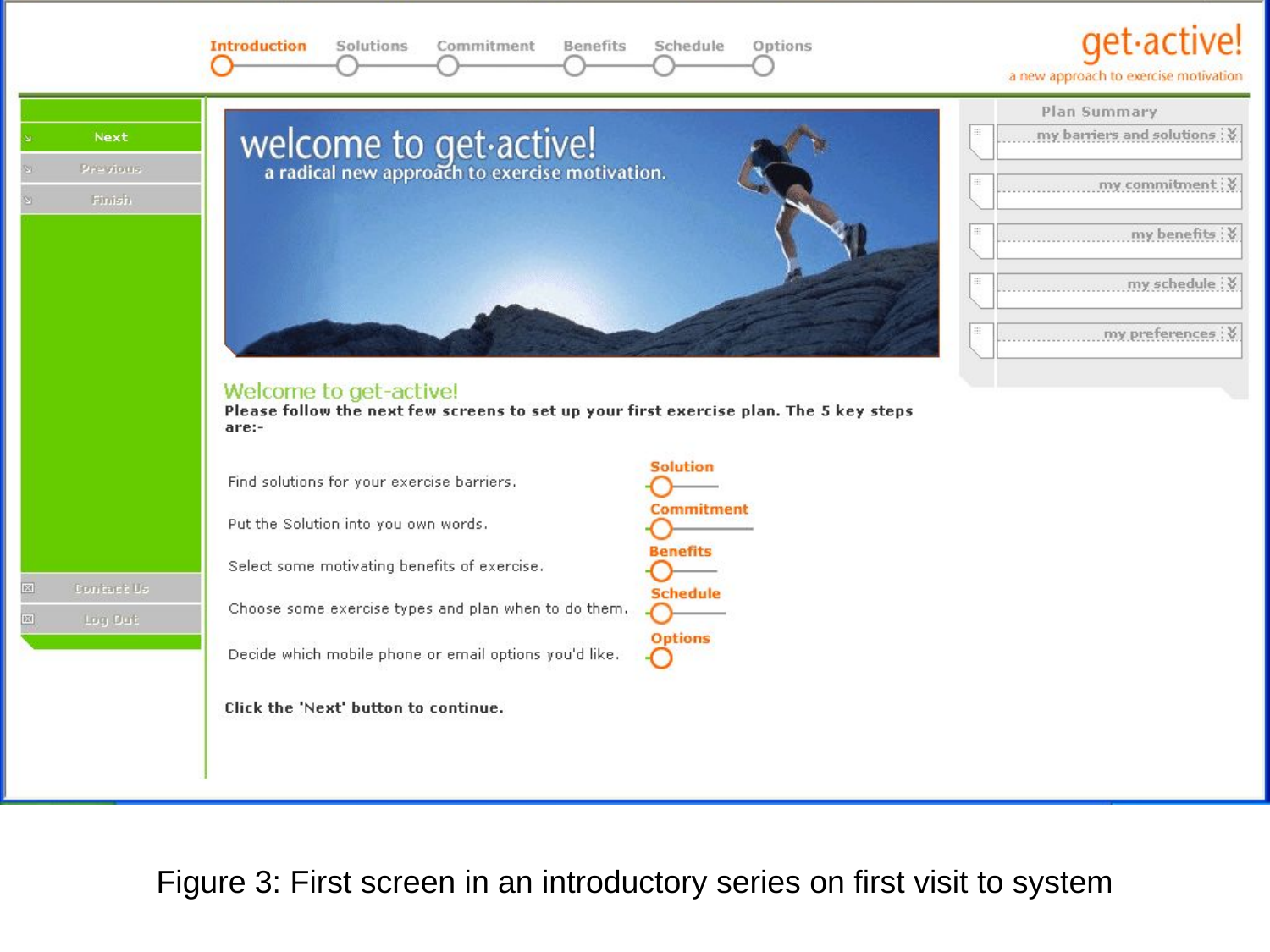

Figure 3: First screen in an introductory series on first visit to system

## Slide 3
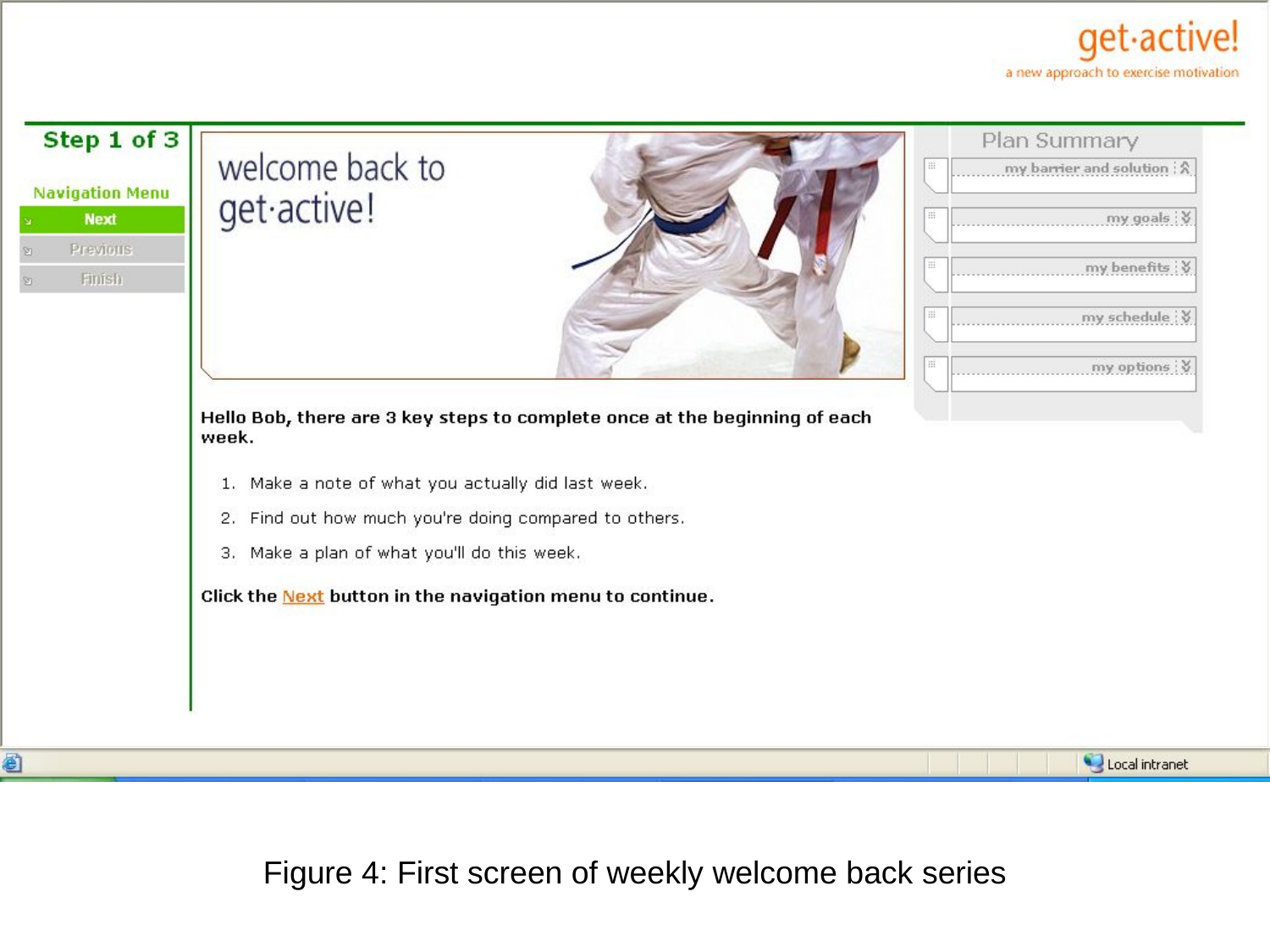

Figure 4: First screen of weekly welcome back series

## Slide 4
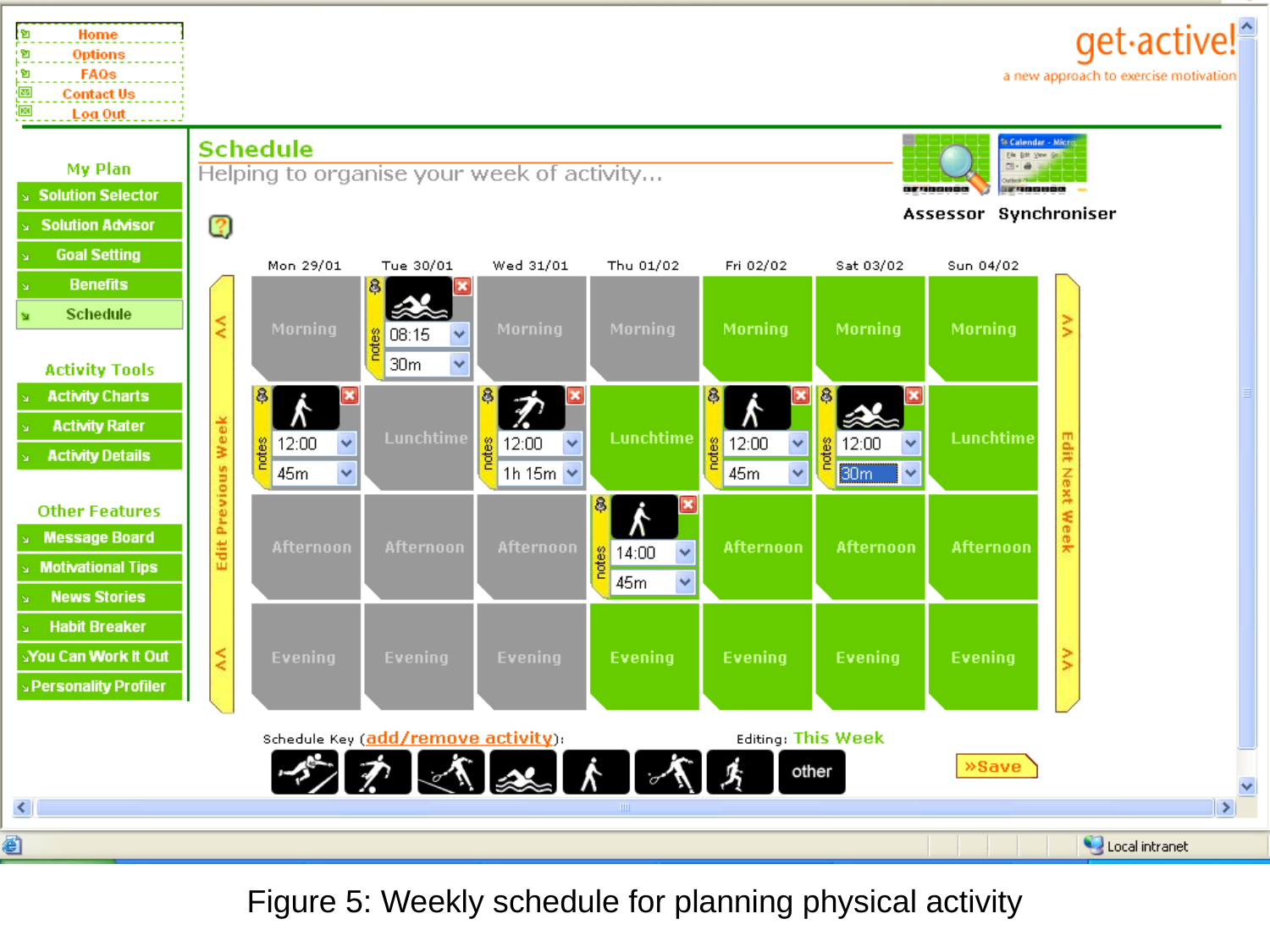

Figure 5: Weekly schedule for planning physical activity

## Slide 5
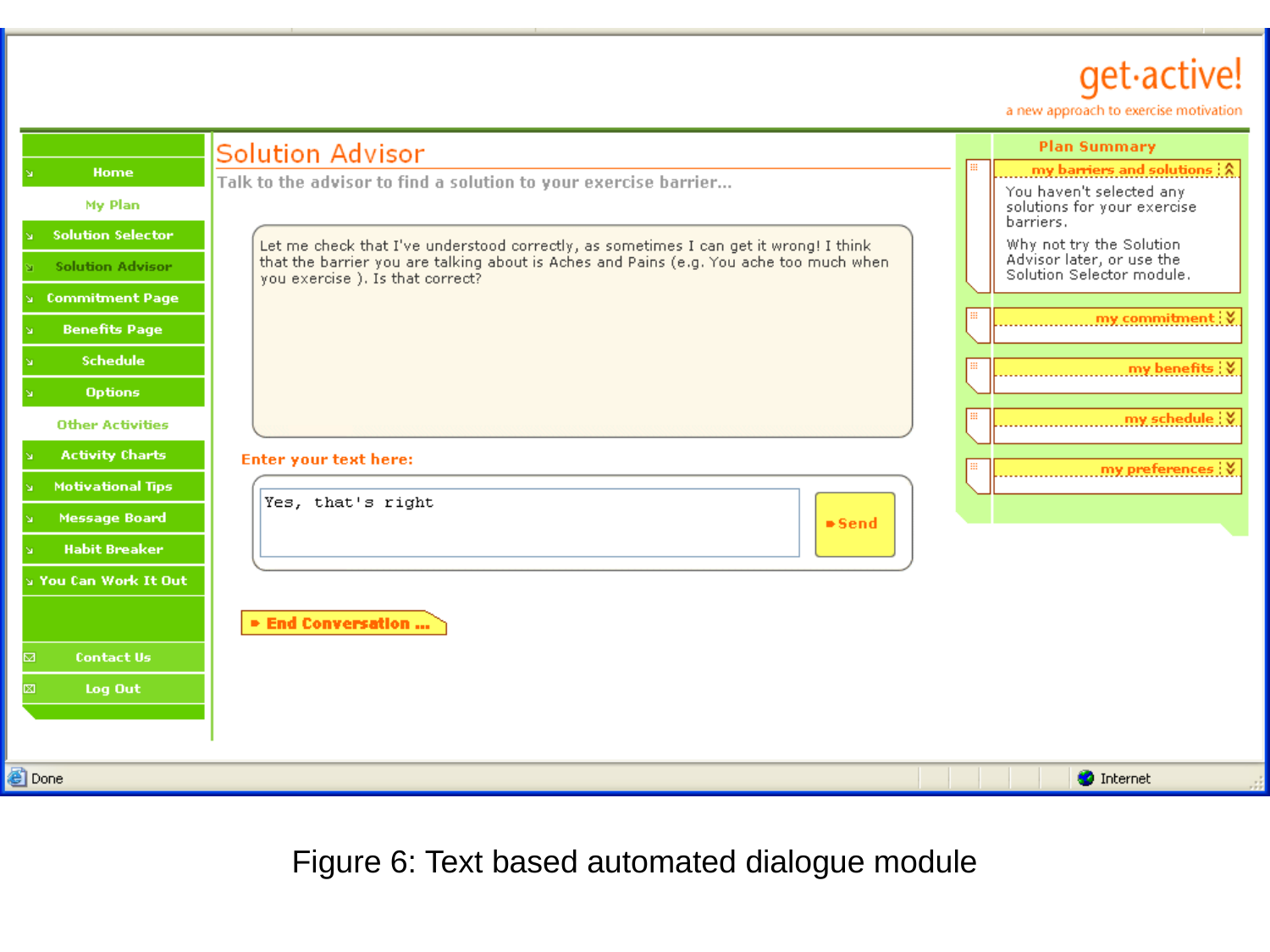

Figure 6: Text based automated dialogue module

## Slide 6
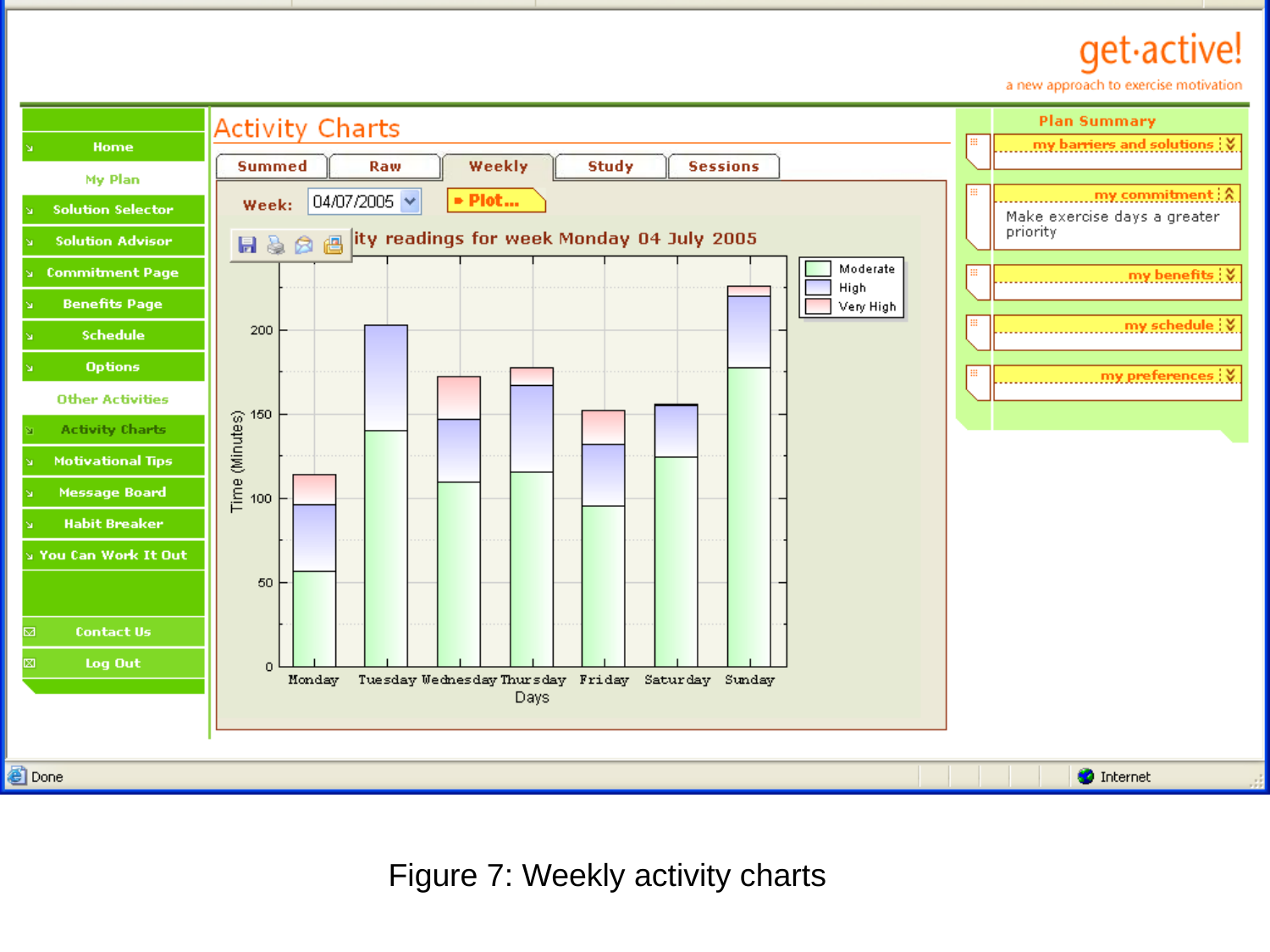

Figure 7: Weekly activity charts
